# Polarized Raman Study of First-Order Phonons in Self-Flux Grown Single-Crystalline WTe_2_

**DOI:** 10.3390/nano14151256

**Published:** 2024-07-27

**Authors:** Peter M. Rafailov, Dimitre Dimitrov, Daniela Kovacheva, Vera Marinova

**Affiliations:** 1 “G. Nadjakov” Institute of Solid State Physics, Bulgarian Academy of Sciences, 1784 Sofia, Bulgaria; rafailov@issp.bas.bg (P.M.R.); dzdimitrov@issp.bas.bg (D.D.); 2 Institute of Optical Materials and Technologies, Bulgarian Academy of Sciences, 1113 Sofia, Bulgaria; 3 Institute of General and Inorganic Chemistry, Bulgarian Academy of Sciences, 1113 Sofia, Bulgaria; didka@svr.igic.bas.bg

**Keywords:** tungsten ditelluride, polarized Raman spectroscopy, XRD, phonons, self-flux, crystal growth

## Abstract

Bulk single crystals of WTe_2_ were grown by the self-flux method and characterized by X-ray diffraction, polarized micro-Raman spectroscopy, and optical microscopy. All methods revealed a high crystalline quality, thus demonstrating the advantages of the growth method used as a starting base for the synthesis of high-quality 2D materials. In each main scattering configuration, we recorded a series of Raman spectra in different sample orientations achieved by rotating the sample around the incident laser beam. In addition to the well-established case of excitation along the c crystal axis, we also applied laser excitation along the a and b axes. Thus, scattering configurations were also realized in the XZ and YZ polarization planes, for which no comparative literature data have yet been established. In these experiments, two new Raman-active phonons with B_2_ symmetry and frequencies of 89 cm^−1^ and 122 cm^−1^ were identified. The obtained experimental data enabled us to derive the magnitude ratios of all three tensor elements of the A_1_ modes and to find their phase differences.

## 1. Introduction

Tungsten ditelluride (WTe_2_), a member of the transition metal dichalcogenides (TMDs) family [1], due to its unique electronic and structural properties, exhibits a rich array of phenomena, including large and anisotropic magnetoresistance [2], unconventional superconductivity [3], and the potential for hosting exotic quantum states [4]. Its intriguing behaviour has sparked intense research interest, driven both by fundamental scientific curiosity and the promise of novel technological applications.

The layered crystal structure of WTe_2_, comprising stacked atomic layers held together by weak van der Waals forces [5], endows it with remarkable two-dimensional characteristics, reminiscent of other TMDs like graphene and molybdenum disulfide (MoS_2_) [6]. However, unlike its counterparts, WTe_2_ exhibits a unique combination of broken inversion symmetry and strong spin-orbit coupling leading to intriguing electronic band structures and topologically non-trivial properties [7].

One of the most striking features of WTe_2_ is its pronounced and unconventional magnetoresistance behaviour, characterized by a colossal and highly anisotropic response to external magnetic fields [8]. This phenomenon has gathered significant attention for its potential in spintronic and quantum computing applications, as well as for its fundamental implications on our understanding of electronic transport in low-dimensional systems.

Furthermore, recent experimental investigations have unveiled evidence of superconductivity in WTe_2_ under specific conditions [9], further adding to its allure as a platform for exploring novel quantum phenomena. The interplay between superconductivity, magnetism, and topology in WTe_2_ presents a fertile ground for studying emergent quantum states and exotic phase transitions.

Synthesizing atomically thin layers of WTe_2_ presents a formidable challenge due to the intrinsic properties of the material and the delicate balance required to achieve monolayer or few-layer flakes with high quality and uniformity [10]. This is especially true for 2D WTe_2_ achieved through the direct conversion of metal and metal-oxide films due to the low reactivity between tellurium and the transition metal tungsten [11]. Techniques such as chemical vapor deposition (CVD) and molecular beam epitaxy (MBE), while promising, often struggle to produce large-area, defect-free 2D samples, limiting their applicability in certain experimental studies and device applications.

In contrast, the synthesis of bulk crystals of WTe_2_ offers distinct advantages in terms of quality and reproducibility [12]. High-temperature methods such as chemical vapor transport (CVT) or flux-growth techniques enable the production of large, high-purity crystals with well-defined structural and electronic properties. While high-quality single crystals can be grown using the CVT [13] method, the self-flux [14] method consistently yields even higher-quality single crystals. In the CVT method, the transport agents can introduce impurities into the crystal. On the other hand, in the self-flux method, the low melting point and high boiling point of elemental Te provide a large temperature window to grow crystals from the melt. Additionally, the absence of a transport agent eliminates a source of impurities. Studies [14] have shown that the residual resistivity ratio (RRR) of flux-grown crystals reaches much higher values compared to those of CVT-grown crystals, implying fewer defects and impurities and leading to an order of magnitude higher magnetoresistance [15]. The self-flux crystal growth method has been successfully used for the synthesis of other TMDs, such as PdSe_2_ [16], WSe_2_, and MoSe_2_ [17,18]. The high-quality bulk crystals grown by this method serve as invaluable platforms for conducting precise measurements and exploring fundamental phenomena, providing insights that complement those obtained from 2D samples.

WTe_2_ has particularly attracted significant interest due to the extremely large magnetoresistance effect in the otherwise diamagnetic WTe_2_ single crystal—a phenomenon not matched in other TMDCs [2]. There is also a remarkably strong dependence of the resistivity on the thickness of WTe_2_ multilayer flakes [19]. Given these properties and the resulting application prospects, a detailed knowledge of the vibrational structure of WTe_2_ is of essential importance.

Several studies have been devoted to the Raman response of crystalline WTe_2_ [19,20,21,22,23,24,25,26]. Due to sample constraints, these studies used only back-scattering experiments from the XY surface with exciting laser beam perpendicular to the layers. Under these conditions, only vibrational modes of A_1_ and A_2_ symmetry could be detected. Here, we report the synthesis of sufficiently large free-standing WTe_2_ single crystals using the self-flux crystal growth method, followed by detailed Raman characterization in all essential polarization geometries without restriction to the XY surface. We detected two new Raman active modes of B_2_ symmetry and obtained some important characteristics of all three elements of the A_1_ Raman tensors.

## 2. Materials and Methods

WTe_2_ single crystals were obtained by a self-flux method using tellurium flux, taking advantage of the very slight solubility [14] of W and WTe_2_ in tellurium. The method is reproducible and yields consistently higher-quality single crystals than are typically obtained via halide-assisted vapor transport methods. In a typical recipe, 9.195 g of W and 230 g of purified Te (99.9999%) were sealed in an evacuated quartz tube. A small amount of quartz wool was added to the tube to act as a filter in order to separate the flux material from the crystals in a later step. The tube was then heated to 825 °C, held there for 24 h, and then cooled at a rate of 2–3 °C/h to 525 °C. At this temperature, the flux was separated from the crystals by inverting the tube. The WT_e2_ crystals were then put in another vacuum-sealed quartz tube and heated to 415 °C in a tube furnace, with WTe_2_ crystals on the hot end and the cold end held at 200 °C. This was done for 2 days in order to separate excess Te from the crystal surfaces via self-vapor-transport and to anneal the crystals. Under these conditions, several lengthy ribbon-like WT_e2_ crystals of mm size were obtained.

X-ray diffraction patterns were collected within the 2θ range from 10 to 80° with a constant step of 0.02° on a Bruker D8 Advance diffractometer (Billerica, MA, USA) with tube CuKα 40 KV, 40 mA. Goniometer radius 217.5 mm, scan type coupled TwoTheta/Theta, 10–80°, step 0.02, counting time 35 s/step, primary Soller slit 4°, divergent slit 0.3°, secondary Soller slit 4°, anti-scattered slit 0.5°, LynxEye detector (Billerica, MA, USA) and detector slit 11.93 mm. Phase identification was performed with the Diffracplus EVA using the ICDD-PDF2 Database (Newtown Square, PA, USA).

The Raman spectra were measured in backscattering geometry using a HORIBA Jobin Yvon Labram HR-visible spectrometer (HORIBA Scientific, Kyoto, Japan) equipped with a Peltier-cooled CCD detector. The 632.8 nm line of a He-Ne laser was used for excitation. The laser beam was focused on a spot of about 1–2 µm in diameter on the sample surface using microscope optics. A diffraction grating of 1800 lines·mm^−1^ was used for dispersing the Raman signal, assuring a spectral resolution better than 1 cm^−1^. The laser power was attenuated below 500 µW to avoid sample overheating. Si standard was used to calibrate the frequency, and the line intensities were determined by fitting them to Voigt profiles.

## 3. Results

### 3.1. X-ray Diffraction Analysis

**Figure 1:**
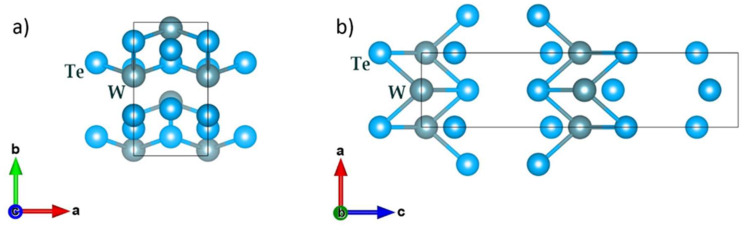
Schematic view of the lattice structure of the WTe2 crystal. The rectangles mark the projections of the elementary cell: (**a**) a-b plane; (**b**) a-c plane.

Tungsten ditelluride (WTe_2_) exhibits a unique crystal structure with an orthorhombic lattice belonging to the Pmn2_1_ space group [27,28,29]. The material possesses a large interlayer spacing and a semi-metallic electronic structure. In its crystalline form, WTe_2_ comprises two-dimensional layers oriented in the (0, 0, 1) direction [2]. Each layer consists of distorted edge-sharing WTe_6_ pentagonal pyramids, with W^4+^ atoms bonded to six Te^2−^ atoms, as depicted in Figure 1 [13].

The crystal structure of WTe_2_ contrasts with other transition metal dichalcogenides (TMDs) like MoS_2_, which typically have a hexagonal lattice. In WTe_2_, the triple-layer Te-W-Te atomic planes crystallize in an orthorhombic, distorted-1T structure with C_2v_ symmetry [14]. Although the chalcogenides around the metal exhibit octahedral coordination, the tungsten atoms are slightly offset from the centre of the Te octahedron [14]. This off-centring results in slightly buckled W-W zigzag chains along the a-axis of the unit cell, contributing to pronounced in-plane anisotropy [13,21].

**Figure 2:**
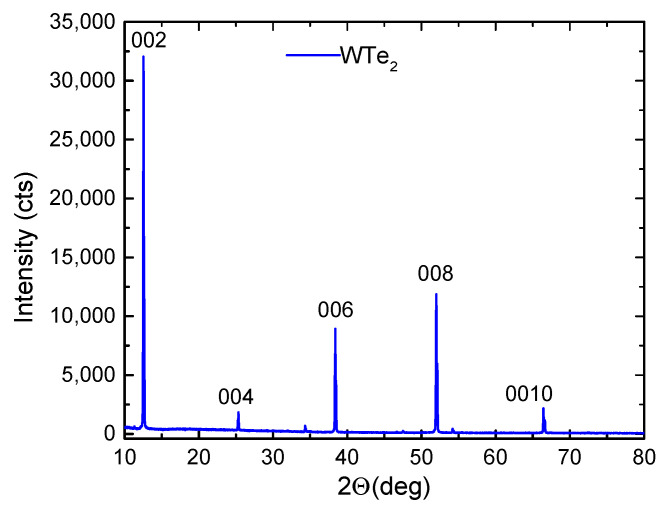
XRD spectrum of the studied WTe_2_ single crystal.

The X-ray diffraction pattern of the obtained WTe_2_ crystal aligned along the (001) plane is shown in Figure 2. XRD was performed at room temperature using a Bruker D8 Advance diffractometer. The observed XRD peaks correspond, from left to right, to (00l) with l = 2, 4, 6, 8, and 10, respectively. The calculated lattice constants are as follows: a = 3.482 Å, b = 6.274 Å, and c = 14.049 Å. From the diffraction scans, we only observed peaks consistent with the (001) family from the Td-WTe_2_ phase based on the powder diffraction file reference 98-007-3323 (WTe_2_). The lack of any additional diffraction peaks indicates the c-axis alignment of the crystal.

### 3.2. Raman Analysis

As pointed out in Ref. [21], usually, a well-defined edge is naturally formed after exfoliation of a few layers of WTe_2_ due to the small cleaving energy along the a-axis (i.e., the direction along the W-W chains). Thus, in our ribbon-like crystals, the edges along the ribbons coincide with the a-axis marking the X direction. The b-axis (i.e., Y direction) is the other main axis lying in the ribbon plane being perpendicular to the edges, and the c-axis (Z direction) is perpendicular to the W-Te layers, i.e., to the ribbon plane.

The unit cell of bulk WTe_2_ contains two W atoms and four Te atoms. According to the C_2v_ symmetry, the irreducible representations of the optical phonons at the center of the Brillouin zone (Γ point) are

G_bulk_ = 11A_1_ + 6A_2_ + 5B_1_ + 11B_2_, (1)

Due to the low symmetry, all of the vibrational modes are Raman active, and the 11A_1_, 5B_1_, and 11B_2_ modes are infrared active.

The pertinent Raman tensors for the C_2v_ symmetry group are the following [21]:

(2) $$A1:\left[\begin{array}{ccc} a & 0 & 0\\ 0 & b & 0\\ 0 & 0 & c \end{array}\right];A2:\left[\begin{array}{ccc} 0 & d & 0\\ d & 0 & 0\\ 0 & 0 & 0 \end{array}\right];B1:\left[\begin{array}{ccc} 0 & 0 & e\\ 0 & 0 & 0\\ e & 0 & 0 \end{array}\right];and\;B2:\left[\begin{array}{ccc} 0 & 0 & 0\\ 0 & 0 & f\\ 0 & f & 0 \end{array}\right],$$

The scattering intensity of a particular mode with Raman tensor α is given by

I ~ |*e*_i_. α. *E*_s_|^2^, (3)

where the unit vectors *e*_i_ and *e*_s_ denote the polarization of the incident and scattered light, respectively. For the polarized Raman measurements, we use the notations *X* (100), *Y* (010), and *Z* (001) for the main crystal axes. The applied scattering configurations are labelled by Porto notations.

**Figure 3:**
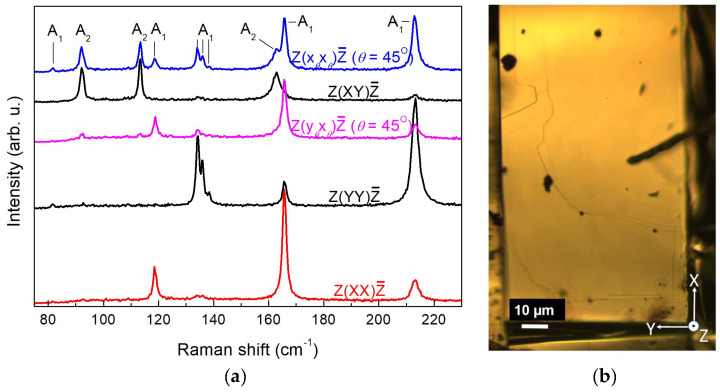
(**a**) Raman spectra of WTe_2_ single crystal with exciting laser beam along Z. (**b**) Optical micrograph from the vicinity of the examined sample spot (smooth surface from a ribbon plane) with indication of the main crystallographic axes.

**Figure 4:**
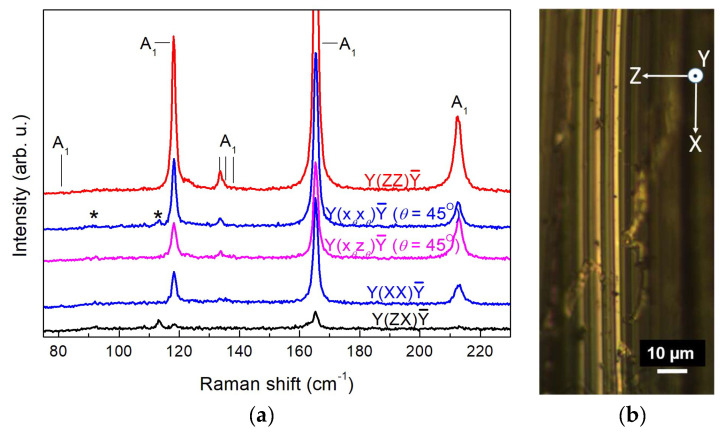
(**a**) Raman spectra of WTe_2_ single crystal with exciting laser beam along Y. Symmetry-forbidden A_2_ lines are marked with asterisks. (**b**) Optical micrograph from the vicinity of the examined sample spot (ribbon edge) with indication of the main crystallographic axes.

**Figure 5:**
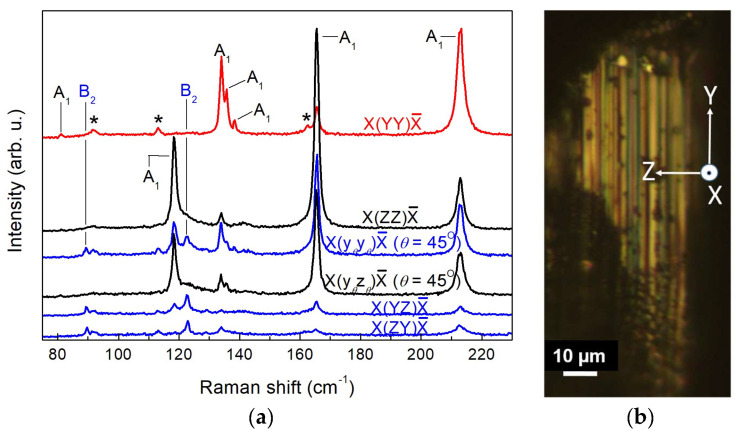
(**a**) Raman spectra of WTe_2_ single crystal with exciting laser beam along X. Symmetry-forbidden A_2_ lines are marked with asterisks. (**b**) Optical micrograph from the vicinity of the examined sample spot (ribbon tip) with indication of the main crystallographic axes.

To complete the mode assignment for the Wte_2_ crystal, we measured Raman spectra as a function of the rotation angle *θ* by rotating the sample around the exciting beam in its polarization plane. All three mutually perpendicular planes, XY, XZ, and YZ, were used as beam-polarization planes, *θ* being the angle between the initial axis (X for the XY and XZ planes and Y for the YZ pane) and the actual polarization direction of the exciting beam. This approach utilising the differences in the transformation properties of phonons of different symmetries has proven to be helpful in the confirmation of phonon symmetry [30]. Although only the XY plane of the Wte_2_ crystal is readily accessible for micro-Raman measurements due to its ribbon-like shape, we performed a search for clean and smooth edges and tips of such ribbons and thus were able to identify a couple of such spots with (010) and (100) orientation. Polarized rotation-angle (*θ*) dependent Raman measurements were carried out in parallel and perpendicular polarization. For *θ*-dependent configurations in perpendicular polarization, x*_θ_*y*_θ_*, x*_θ_*z*_θ_*, and y*_θ_*z*_θ_* denote orthogonal *θ*-dependent directions of incident and scattered radiation in the XY, the XZ, and the YZ plane, respectively. For rotation in parallel polarization, the transition from the initial to the final polarization is marked. For instance, Y(XX→ZZ)Ȳ indicates a gradual transition from Y(XX)Ȳ to Y(ZZ)Ȳ through rotation about the Y axis. Vertically stacked rotation-angle dependent spectra are shown in Figure 3, Figure 4 and Figure 5 for selected scattering configurations in the XY, the XZ, and the YZ planes, respectively. The theoretically predicted dependence of the Raman intensity (Equation (3)) on the angle *θ* for the examined configurations is given in Table 1. It should be pointed out that the experimental accuracy of measurements in the XZ and YZ polarization planes was inevitably lower than that in the XY plane for a number of reasons: (i) it is more difficult to achieve precise orientation of the sample; (ii) the orientational dependence of the Raman intensity is impacted by edge effects originating from the layered crystal structure; (iii) larger vulnerability to chemical degradation at edges and tips of the ribbon-like Wte_2_ crystals. These regions are preferentially attacked by air oxygen, and besides the deterioration of the surface, the Raman spectra may be burdened with TeO_2_ features [19], traces of which are discernible in Figure 4a and Figure 5a around 125 and 142 cm^−1^. Nevertheless, the good crystal quality of the samples enabled us to acquire mutually consistent data from all three polarization planes.

The spectra exhibit sharp lines with a well-defined polarization dependence. The well-studied 7 A_1_ lines (80, 118, 133, 135, 137.5, 165, and 212 cm^−1^) and 3 A_2_ lines (91.5, 112.5, and 162 cm^−1^) in the main range of WTe_2_ vibrations are clearly recognizable. Their line widths (full width at half maximum (FWHM)) are given in Table 2, and almost all of them are below 2 cm^−1^. Although FWHM data are rarely reported in the literature, the line sharpness established in the present study, along with the clean X-ray diffraction spectrum in Figure 2, clearly evidences a high-quality single-crystalline material, thus demonstrating the advantages of the self-flux crystal growth method used.

**Figure 6:**
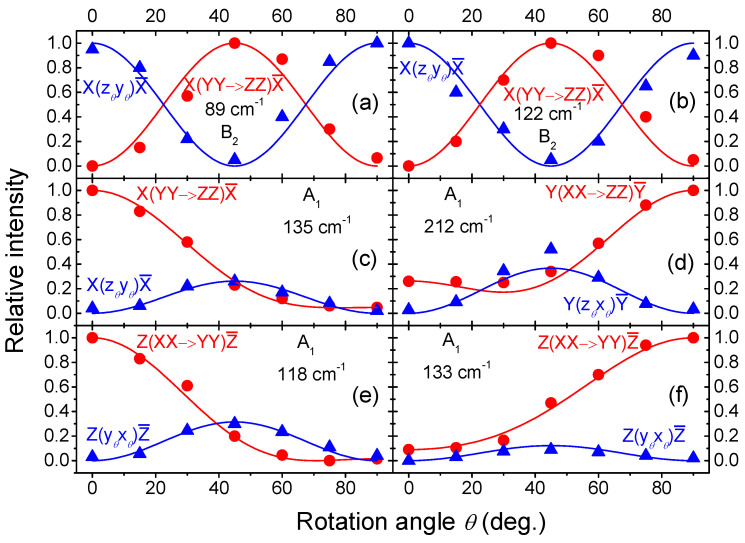
Angular dependence of the relative intensity of WTe_2_ Raman lines in parallel polarization (red circles) and perpendicular polarization (blue triangles) (see text and Table 1). The polarization configurations are indicated in the plots. Panels (**a**,**b**): B_2_ modes at 89 and 122 cm^−1^. Panels (**c**–**f**): A_1_ modes at 135, 212, 118 and 133 cm^−1^, respectively. The solid lines are graphs of the pertinent theoretically predicted functional dependencies.

Comparing Figure 3, Figure 4 and Figure 5, we find two new lines at 89 and 122 cm^−1^ that obviously correspond to Raman-active modes detectable only upon excitation along the X-axis. The angular dependence of the scattering intensity normalized to the maximum intensity for each of these lines is plotted in Figure 6a,b, respectively. The pertinent theoretical curves obtained from the expressions in Table 1 by means of the extrema in the measured line intensities are also plotted in Figure 6a,b for comparison. From Figure 6a,b and Table 1, it straightforwardly follows that the two newly detected modes at 89 and 122 cm^−1^ should be assigned as B_2_ modes. Their frequencies agree well with theoretical modelling results of the vibrational structure of WTe_2_ [19,22].

Additionally, the acquired data enable us to find important parameters of the Raman tensors of the A_1_ modes. WTe_2_ is an opaque semiconductor material with a band structure implying significant absorption in the visible region. The absorption properties of such a material are reflected in the imaginary part of its dielectric function and, hence, also in the imaginary part of its Raman tensor elements, which are proportional to the partial derivatives of the dielectric function along the phonon’s vibrational directions [31,32]. They can be written as *r* = |*r*|·exp(*iϕ_r_*), where *r* represents all Raman tensor elements from Equation (2) [31,33]. It is seen that the three A_1_ tensor elements in the main diagonal can have different phases (*ϕ_a_*, *ϕ_b_*, and *ϕ_c_*) that do not necessarily coincide with each other. This impacts the A_1_ Raman intensity in scattering geometries involving more than one tensor element [34]. The phases of the tensor elements of modes A_2_, B_1_, and B_2_ do not matter for their Raman intensity since it depends only on the magnitudes of these tensor elements in each scattering geometry (see Table 1). It is thus important to know the phase differences between the tensor elements of the A_1_ modes.

Using the complex representation of the A_1_ tensor elements *a* and *b*, one obtains from Table 1 the following expressions [31] for the *θ*-dependent Raman intensity *I*_par_ in parallel polarization (Z(x*_θ_*x*_θ_*)$\bar{Z}$) and *I*_perp_ in perpendicular polarization (Z(x*_θ_*y*_θ_*)$\bar{Z}$) in the XY polarization plane:

(4) $$I_{par}\propto {\left|a\right|}^{2}{\left(\cos\theta \right)}^{4}+{\left|b\right|}^{2}{\left(\sin\theta \right)}^{4}+2\left|a\right|\left|b\right|{\left(\sin\theta \right)}^{2}{\left(\cos\theta \right)}^{2}\cos\phi_{ab},$$

(5) $$I_{perp}\propto \left({\left|a\right|}^{2}+{\left|b\right|}^{2}-2\left|a\right|\left|b\right|\cos\phi_{ab}\right){\left(\sin\theta \right)}^{2}{\left(\cos\theta \right)}^{2},$$

where *ϕ_ab_* = *ϕ_b_* − *ϕ_a_* is the phase difference between the tensor elements *a* and *b*. For *θ* = 45° these expressions become:

(6) $$I_{par}\propto \frac{1}{4}\left({\left|a\right|}^{2}+{\left|b\right|}^{2}+2\left|a\right|\left|b\right|\cos\phi_{ab}\right),$$

(7) $$I_{perp}\propto \frac{1}{4}\left({\left|a\right|}^{2}+{\left|b\right|}^{2}-2\left|a\right|\left|b\right|\cos\phi_{ab}\right)$$

Together with the measured intensities in XX and YY polarization the Equations (6) and (7) form an over-determined equation system, from which the ratio of the tensor element magnitudes |*a*|/|*b*| and their phase difference *ϕ_ab_* can be found. From analogous equation systems for the remaining two polarization planes XZ and YZ, the ratios |*a*|/|*c*| and |*b*|/|*c*| and the phase differences *ϕ_ac_* and *ϕ_bc_* can also be found, respectively. Finally, the sum rule connecting the three phase differences *ϕ_ab_ + ϕ_bc_ + ϕ_ca_* = 0 provides the possibility for an additional consistency check of the results. However, due to experimental inaccuracies, Equations (6) and (7) and their analogous pairs for the other scattering geometries turn out to be not perfectly compatible for every A_1_ mode. Therefore, in order to obtain more accurate results and to utilize the acquired *θ*-dependent data, we used the three equation systems only to find the ratios |*a*|/|*b*|, |*a*|/|*c*| and |*b*|/|*c*| and to obtain normalized values for |*a*|, |*b*|, and |*c*|. Then, each of the three pairs of *θ*-dependent datasets obtained in the three mutually perpendicular polarization planes was fitted with the pertinent pair of equations analogous to Equations (4) and (5) to extract the phase differences. Thus, the obtained complete results for the A_1_ modes are summarized in Table 2. It can be seen that the A_1_ tensor element magnitudes are strongly anisotropic in accordance with the significant lattice anisotropy. Still, the A_1_ phonon at 118 cm^−1^ represents a special case with its extremely large anisotropy in the YZ plane. Here, |*b*|<<|*c*| and, therefore, the pertinent *θ*-dependent datasets are less sensitive [34] to the phase difference *ϕ_bc_*. Therefore, for this phonon, we derived the cos *ϕ_bc_* value (−0.5) in Table 2 from the sum rule requiring the three phase differences to add up to zero instead of using the angle-dependent intensity data fit which rather points to cos *ϕ_bc_* = −1. To the best of our knowledge, analogous results are available in the literature only for the XY polarization plane [21], and only one study reported partial results for the three A_1_ lines at 133, 165, and 212 cm^−1^ from other polarization planes [33].

Four different cases are selected for an illustration in Figure 6. The angular dependence of Raman intensity normalized to the maximum intensity for each selected combination of A_1_ line and scattering geometry is plotted in Figure 6c–f. The pertinent theoretical curves constructed using the obtained tensor-element magnitudes and phase difference values are also added to the plots for comparison. Panels Figure 6d,e depict two cases where the phase difference is between π/2 and π, and for *θ* = 45°, the scattering intensity in perpendicular polarization surpasses that in parallel polarization. Panel Figure 6c shows a case with *ϕ_bc_* ≈ π/2, in which the Raman intensities for *θ* = 45° in parallel and perpendicular polarization are equal. A case with no phase difference is shown in panel Figure 6f with markedly low scattering intensity in perpendicular polarization.

The present results can be applied for the identification of the crystallographic orientation of edges of crystalline WTe_2_ layers and flakes, which is considered very difficult to achieve [33]. A comparison of the behaviour of the A_1_ lines at 118 and 165 cm^−1^ for *θ* = 45° in Figure 4a and Figure 5a reveals that for both lines *I*_par_ > *I*_perp_ with laser excitation along the Y axis and again for both lines *I*_par_ < *I*_perp_ with laser excitation along the X axis. Thus, comparing polarized Raman spectra at laser polarization, making an angle of 45° with the examined edge, (100), and (010) orientations can be distinguished from each other. More broadly, our results provide a foundation for Raman monitoring in all three dimensions of the effects of WTe_2_ sample thinning, which has previously been studied only through backscattering from the basal (XY) plane [23].

## 4. Conclusions

Bulk single crystals of WTe_2_ were successfully grown using the self-flux method and confirmed through X-ray diffraction and polarized micro-Raman spectroscopy. The crystals were investigated in all relevant polarization geometries, allowing us to identify the phonons with a measurable Raman response and determine the ratios between the magnitudes of the tensor elements for the fully symmetric Raman-active phonons of the A_1_ type. Two new Raman-active phonons with B_2_ symmetry and frequencies of 89 cm^−1^ and 122 cm^−1^ have been identified. From azimuthal-angle-dependent Raman spectra in the three mutually perpendicular polarization planes XY, XZ, and YZ, we were also able to estimate the phase differences of the three complex tensor elements of the A_1_-type phonons. This demonstrates the good crystal quality and the prospects of the growth method used as a starting base for the synthesis of high-quality 2D materials.

## Figures and Tables

**Table 1:** Theoretical dependence of the Raman intensities of WTe_2_ phonons on the rotational angle *θ* in the various scattering configurations.

Mode Symmetry	Z(x*_θ_*x*_θ_*)$\bar{Z}$ (XX→YY)	Z(x*_θ_y_θ_*)$\bar{Z}$ (XY→YX)	Y(x*_θ_*x*_θ_*)$\bar{Y}$ (XX→ZZ)	Y(x*_θ_*z*_θ_*)$\bar{Y}$ (XZ→ZX)
A_1_	|*a*·cos^2^*θ* + *b*·sin^2^*θ*|^2^	(|*a* − *b*|^2^/4)·sin^2^2*θ*	|*a*·cos^2^*θ* + *c*·sin^2^*θ*|^2^	(|*a* − *c*|^2^/4)·sin^2^2*θ*
A_2_	|*d*|^2^·sin^2^2*θ*	|*d*|^2^·cos^2^2*θ*	0	0
B_1_	0	0	|*e*|^2^·sin^2^2*θ*	|*e*|^2^·cos^2^2*θ*
B_2_	0	0	0	0
**Mode** **Symmetry**	**X(y*_θ_*y*_θ_*)$\bar{X}$** **(YY→ZZ)**	**X(y*_θ_*z*_θ_*)$\bar{X}$** **(YZ→ZY)**		
A_1_	|*b*·cos^2^*θ* + *c*·sin^2^*θ*|^2^	(|*b* − *c*|^2^/4)·sin^2^2*θ*		
A_2_	0	0		
B_1_	0	0		
B_2_	|*f*|^2^·sin^2^2*θ*	|*f*|^2^·cos^2^2*θ*		

**Table 2:** Important spectral parameters of the A_1_ Raman active modes of WTe_2_. The cos *ϕ*_bc_ value for the A_1_ mode at 118 cm^−1^ is marked with an asterisk because it was derived from the values of cos *ϕ*_ab_ and cos *ϕ*_ac_ instead of fitting angle-dependent intensity data (see text).

Experimental Frequency (cm^−1^)	80	118	133	135	137.5	165	212
Full width at half maximum (cm^−1^)	1.5	1.5	1.5	1.5	1.5	1.8	2.7
Ratio of magnitudes of Raman tensor elements |*a*|:|*b*|:|*c*|	1.5 : 2 : 1	6 : 1 : 13	1 : 4 : 2	1.5 : 5 : 1	1 : 3 : 1.5	2.5 : 1 : 4	1 : 3 : 2
cos *ϕ*_ab_	1	−1	1	1	0.3	0.3	0.9
cos *ϕ*_ac_	−0.3	0.5	0.3	0	−0.5	0.5	−0.2
cos *ϕ*_bc_	−0.3	−0.5 *	0.3	0	0.7	−0.7	0.2

## Data Availability

Data are contained within the article.
